# Artificial intelligence–assisted cancer diagnosis improves the efficiency of pathologists in prostatic biopsies

**DOI:** 10.1007/s00428-023-03518-5

**Published:** 2023-02-21

**Authors:** Catarina Eloy, Ana Marques, João Pinto, Jorge Pinheiro, Sofia Campelos, Mónica Curado, João Vale, António Polónia

**Affiliations:** 1 Pathology Laboratory, Institute of Molecular Pathology and Immunology of the University of Porto (Ipatimup), Porto, Portugal; 2 i3S - Instituto de Investigação E Inovação Em Saúde, Porto, Portugal; 3 Faculty of Medicine, University of Porto, Porto, Portugal; 4 Serviço de Anatomia Patológica, Centro Hospitalar Universitário de São João, Porto, Portugal; 5 Serviço de Anatomia Patológica, Hospital Pedro Hispano – Unidade Local de Saúde de Matosinhos, Matosinhos, Portugal

**Keywords:** Artificial intelligence, Prostate cancer, Computational pathology, Digital pathology

## Abstract

Paige Prostate is a clinical-grade artificial intelligence tool designed to assist the pathologist in detecting, grading, and quantifying prostate cancer. In this work, a cohort of 105 prostate core needle biopsies (CNBs) was evaluated through digital pathology. Then, we compared the diagnostic performance of four pathologists diagnosing prostatic CNB unaided and, in a second phase, assisted by Paige Prostate. In phase 1, pathologists had a diagnostic accuracy for prostate cancer of 95.00%, maintaining their performance in phase 2 (93.81%), with an intraobserver concordance rate between phases of 98.81%. In phase 2, pathologists reported atypical small acinar proliferation (ASAP) less often (about 30% less). Additionally, they requested significantly fewer immunohistochemistry (IHC) studies (about 20% less) and second opinions (about 40% less). The median time required for reading and reporting each slide was about 20% lower in phase 2, in both negative and cancer cases. Lastly, the average total agreement with the software performance was observed in about 70% of the cases, being significantly higher in negative cases (about 90%) than in cancer cases (about 30%). Most of the diagnostic discordances occurred in distinguishing negative cases with ASAP from small foci of well-differentiated (less than 1.5 mm) acinar adenocarcinoma. In conclusion, the synergic usage of Paige Prostate contributes to a significant decrease in IHC studies, second opinion requests, and time for reporting while maintaining highly accurate diagnostic standards.

**Supplementary information:**

The online version contains supplementary material available at 10.1007/s00428-023-03518-5.

## Introduction

Prostate cancer is a frequent disease with important consequent morbidity and mortality among male patients [1]. The diagnosis of prostate cancer rests in performing a core needle biopsy (CNB) of the prostate in patients with elevated blood prostate-specific antigen (PSA) and/or abnormal digital rectal examination. Depending on the biopsy modality and the number of cores obtained, the morphological examination of the prostatic tissue by the pathologist may be a time-consuming task that requires steady concentration to detect minimal alterations of the glandular architecture as well as cellular atypia [2]. Besides cancer detection and classification, a structured report has to include tumor grading and quantification to guide patient management [3]. Classical quality control measures such as second opinion requests or complementary immunohistochemistry (IHC) studies help in increasing cancer detection, decreasing error, and lowering the levels of interobserver variability [4, 5]. These classical measures have a significant impact on the time needed for reporting, ultimately leading to a delay in decisions which impact patient management.

Technological advances in modern digital pathology allow the production of high-quality whole-slide images (WSIs) that are progressively being assumed as the new standard in diagnosis [6, 7]. High-quality WSIs are also the perfect substrate for computational analysis, namely the application of artificial intelligence (AI) tools, that have demonstrated their value in a substantial number of cancer models [8–10], including prostatic adenocarcinoma [11–14].

Paige Prostate is a clinical-grade AI tool created as described by Campanella et al. [14] which is designed to assist the pathologist in the diagnosis of prostate cancer. Paige Prostate is a deep learning tool trained using a weakly supervised approach of convolutional neuronal network (CNNs) [14] that runs in the proprietary, Food and Drug Administration (FDA)-approved viewer called FullFocus. Page Prostate Detect is a binary classificator and produces a slide-level score, either benign or suspicious for harboring adenocarcinoma, also authorized by the FDA. Paige Prostate Grade & Quantify is designed to evaluate the Gleason score, primary and secondary Gleason patterns, as well as cancer length and percentage in each CNB. In the past 2 years, several studies have demonstrated Paige Prostate as a good prescreening tool as well as a reliable second reader [11], contributing to a significant decrease in diagnostic time and increment in diagnostic accuracy [12, 15].

The work herein described intents to challenge Paige Prostate software in the setting of a fully digital laboratory, comparing the diagnostic performance of 4 pathologists diagnosing prostatic CNB specimens unaided and, in a second phase, assisted by Paige Prostate.

## Materials and methods

### Cohort selection

A cohort of consecutive prostate CNBs primarily reported with the support of immunohistochemistry was retrieved from the digital archives of the Pathology Laboratory of Ipatimup from March 2021 to September 2021 (7 months). The cohort included 105 WSIs from the corresponding hematoxylin–eosin (HE) glass slides that were obtained from formalin-fixed paraffin-embedded prostate CNBs collected from 41 patients. All slides of the cases were included.

At the time of primary diagnosis, an IHC double staining was performed in 3-µm-thick sections of all 105 paraffin blocks, with p63 mouse polyclonal primary antibody (Ventana anti-p63 (4A4); Ventana Medical Systems, Inc., Tucson, AZ, USA) and racemase/p504s rabbit monoclonal primary antibody (Vitro anti-p504s (13H4); Vitro Master Diagnostica, Seville, Spain). The OptiView DAB IHC Detection Kit (Ventana Medical Systems, Inc., Tucson, AZ, USA) was used. The entire procedure was carried out on an automated staining system (Ventana BenchMark XT Staining System; Ventana Medical Systems, Inc., Tucson, AZ, USA) according to the manufacturer’s instructions. Appropriate positive and negative controls for both primary antibodies were used in every slide.

All slides (H&E and IHC) were scanned with the Pannoramic 1000 DX scanner (3DHISTECH, Ltd., Budapest, Hungary) at 20 × magnification, with a protocol previously validated for primary diagnosis (pixel scale of 0.243 µm/pixel) [6].

### Evaluation of WSIs

This study followed a multi-reader, multi-case design with modality crossover, whereby the same pathologists read the same cases twice, first unaided (phase 1) and then aided by Paige Prostate after a washout period of at least 2 weeks (phase 2). In phase 1, four pathologists evaluated all H&E-stained WSIs (from now on designated as cases) using the CaseViewer (3DHISTECH, Ltd., Budapest, Hungary) in a 32-in. monitor (Sharp PN-K322BH, 3840 × 2160 resolution in dots—QFHD) as for routine diagnosis [6]. Although all WSIs had a paired IHC slide with double-staining p63 and racemase, pathologists were instructed to evaluate the IHC slide only if they would have requested it in clinical practice. Additionally, in cases where the pathologists would have needed a second opinion, they were able to consult the original report, which represented a constant second opinion for all.

After the washout period, in phase 2, the same four pathologists re-evaluated the same cases using the FullFocus viewer (FDA approved; CE-in vitro diagnostic (IVD)) and assisted by Paige Prostate (Paige, New York, NY, USA), maintaining the same conditions described above. The software was comprised of Paige Prostate Detect (FDA cleared; CE-IVD) and Paige Prostate Grade & Quantify (CE-IVD) tools. The first tool assists pathologists in the detection of suspicious foci for cancer, providing an active focus of interest and suspicious tissue heatmap, and the second tool provides a Gleason score together with percentages of each Gleason pattern present on that WSI, as well as total tumor percentage and total tumor length (in millimeters with two decimal places).

All parameters evaluated were recorded in both phases manually in a prefilled Excel sheet (Microsoft, Redmond, WA, USA), which acted as a simulated reporting tool. The reading pathologists had no time constrains during the evaluation of the cases, and the time of analysis was measured from the opening of the WSI until the case was reported on Excel. The following parameters were recorded per each case in each phase: diagnosis of cancer (yes or no), cancer type, grade group (GrG) (1–5 or not applicable), cribriform pattern (present or absent), intraductal carcinoma (present or absent), perineural invasion (present or absent), number of fragments with cancer, linear tissue size (in mm), linear cancer size (in mm), request for IHC (yes or no), request for second opinion (yes or no), total agreement with the software (yes or no), and time for reporting (in seconds). At the end, there was the possibility of adding additional findings, such as the presence of atypical small acinar proliferation (ASAP).

The ground truth (GT) was established as total agreement between the four pathologists who evaluated the cases (AM, JgP, JP, and SC) or, otherwise, the consensus between two additional independent pathologists through a common WSI session with access to IHC studies and Paige Prostate results (CE and AP). The pathologists (P1 to P4) that evaluated all the cases are generalist pathologists with 2 years, 4 years, 4 years, and 9 years of practice, respectively. The independent pathologists are also generalist pathologists with 9 years and 12 years of practice.

### Statistical analysis

Statistical analyses were performed using the Statistical Package for Social Sciences (SPSS) version 27.0 for Windows (IBM). Pearson’s chi-squared (*χ*^2^) test and the McNemar (MN) test were used for comparison of qualitative variables, and the Mann–Whitney (MW) test, the Wilcoxon test, and the Kruskal–Wallis (KW) test were used for comparison of quantitative variables. The level of significance was set at *p* < 0.05. Concordance rates were evaluated with simple (diagnostic concordance) and quadratic weighted (GrG concordance) kappa statistics to penalize discordances with higher clinical impact. The Landis and Koch classification was used to interpret the values: no agreement to slight agreement (< 0.20), fair agreement (0.21–0.40), moderate agreement (0.41–0.60), substantial agreement (0.61–0.80), and excellent agreement (> 0.81).

The authors used the Altman-Bland analysis to assess the agreement between measurements of cancer sizes. The *x*-axis represents the mean of the measurements, and the *y*-axis shows the difference between the measurements for each case. Altman-Bland plots display the mean difference (solid line) and 95% agreement limits (dashed lines). If there is high agreement between measurements, the mean difference is expected to be centered around zero, with a narrow agreement limit.

## Results

The cohort characteristics are summarized in Table 1 and included prostate CNBs from 41 men with a median age of 69 years (range: 50–85 years old) at the time of diagnosis. Of the 105 slides, 66 (62.86%) were benign and 39 (37.14%) had a diagnosis of cancer, all acinar adenocarcinoma, from 25 men. GrG distribution was as follows: 19 cases for GrG1 (48.72%), 8 cases for GrG2 (20.51%), 5 cases for GrG3 (12.82%), 2 cases for GrG4 (5.13%), and 4 cases for GrG5 (10.26%), and 1 case (2.56%) was not graded due to post-radiation therapy.

**Table 1:** Cohort characteristics

Total patients, *n*	41
Patient age, years (median [P25–P75])	69 [61–74]
Total cases (core needle biopsies)	105
Negative, including atypia, *n* (%)	66 (62.86)
Prostate cancer, *n* (%)	39 (37.14)
Histological type (for prostate cancer only), *n* (%)	
Acinar adenocarcinoma	39 (100)
Grade group, *n* (%)	
1	19 (48.72)
2	8 (20.51)
3	5 (12.82)
4	2 (5.13)
5	4 (10.26)
Not applicable	1 (2.56)

*P25* 25th percentile, *P75* 75th percentile

In phase 1, pathologists had a global diagnostic accuracy for prostate cancer of 95.00% (range: 93.33–97.14%; kappa range: 0.862–0.938) and a mean interobserver diagnostic concordance rate of 94.13% (range: 90.48–98.10%; kappa range: 0.802–0.961) (Table 2 and S1). In phase 2, with the assistance of the software, pathologists had similar global diagnostic accuracy (93.81%; range: 91.43–95.24%; kappa range: 0.823–0.896; MN test: *p* > 0.999) as well as similar mean interobserver diagnostic concordance rate (93.02%; range: 90.48–97.14%; kappa range: 0.802–0.942) (Table 2 and S1). The global diagnostic intraobserver concordance rate between phases was 98.81% (range: 98.10–100%; kappa range: 0.958–1.000).

**Table 2:** Diagnostic accuracy in phases 1 and 2 and diagnostic and grade group intraobserver concordances

	Diagnostic accuracy		*p*	Diagnostic intraobserver concordance, proportion in % (kappa)	Grade group intraobserver concordance, proportion in % (kappa quadratic weighted)
	Phase 1 (%)	Phase 2 (%)			
P1	93.33	91.43	> 0.999^a^	98.10 (0.961)	68.89 (0.844)
P2	94.29	94.29	> 0.999^a^	100 (1.000)	63.42 (0.840)
P3	95.24	94.29	> 0.999^a^	99.05 (0.981)	84.09 (0.954)
P4	97.14	95.24	> 0.999^a^	98.10 (0.958)	80.00 (0.830)
Global	95.00	93.81	> 0.999^a^	98.81 (0.975)	73.94 (0.868)

^a^McNemar test

In phase 1, the average performance of pathologists for diagnosis of prostate cancer was as follows: sensitivity of 0.968 (range: 0.923–1.000), specificity of 0.939 (range: 0.894–1.000), positive predictive value of 0.909 (range: 0.848–1.000), and negative predictive value of 0.982 (range: 0.957–1.000) (Table S2). In phase 2, we observed similar average values: sensitivity of 0.955 (range: 0.897–1.000), specificity of 0.928 (range: 0.879–0.985), positive predictive value of 0.892 (range: 0.826–0.972), and negative predictive value of 0.974 (range: 0.942–1.000) (Table S2).

In phase 1, the mean GrG concordance rate with the ground truth was 80.58% (range: 64.86–94.29%; kappa range: 0.814–0.963) and the mean interobserver GrG concordance rate was 73.39% (range: 57.50–86.11%; kappa range: 0.823–0.942) (Table S3). In phase 2, the mean GrG concordance rate with the ground truth was similar (78.91%; range: 67.57–88.24%; kappa range: 0.791–0.960) as well as the mean interobserver GrG concordance rate (72.03%; range: 64.71–80.00%; kappa range: 0.760–0.938) (Table S3). The global GrG intraobserver concordance rate between phases was 73.94% (range: 63.42–84.09%; kappa range: 0.830–0.954) (Table 2).

There were no significant differences in the detection of cribriform pattern (15.15% vs 13.94%; MN test: *p* = 0.867), intraductal carcinoma (5.99% vs 5.36%; MN test: *p* = 0.842), or perineural invasion (10.18% vs 4.76%; MN test: *p* = 0.061) when comparing phase 1 to phase 2, respectively. Interestingly, in phase 2, pathologists showed a significant reduction (32.35%) in the report of ASAP (34 cases [8.10% in phase 1] and 23 cases [5.48% in phase 2]) (Fisher’s exact test: *p* < 0.001).

Pathologists requested significantly fewer IHC (36.43% vs 45.95%; MN test: *p* < 0.001) and second opinions (7.38% vs 12.14%; MN test: *p* = 0.006) in phase 2 compared with phase 1, respectively (Table 3). This significant reduction in IHC and second opinion requests in phase 2 occurred for both cancer (IHC: 24.72% reduction; second opinion: 37.93% reduction) and negative (IHC: 17.31% reduction; second opinion: 40.91% reduction) cases (Table 3).

**Table 3:** Differences in immunohistochemistry and second opinion requests in both phases

	Phase 1, *n* (%)	Phase 2, *n* (%)	*p*	Reduction from phase 1 to phase 2 (%)
IHC requests				
All cases	193 (45.95)	153 (36.43)	< 0.001^a^	20.72
Cancer cases	89 (57.05)	67 (42.95)	< 0.001^a^	24.72
Negative cases	104 (39.39)	86 (32.58)	< 0.001^a^	17.31
2^nd^ Op requests				
All cases	51 (12.14)	31 (7.38)	< 0.001^b^	39.21
Cancer cases	29 (18.59)	18 (11.54)	0.001^b^	37.93
Negative cases	22 (8.33)	13 (4.92)	< 0.001^b^	40.91

*IHC* immunohistochemistry, *2*^*nd*^* Op* second opinion

^a^Chi-squared test

^b^Fisher’s exact test

Importantly, the median time required for reading and reporting each slide was 139.00 s (P25–P75: 91.00–243.25) in phase 1 and 108.50 s (P25–P75: 73.25–191.75) in phase 2, corresponding to a decrease in 21.94% of the time required (Wilcoxon test: *p* < 0.001). For negative cases, pathologists required 18.41% longer for reporting during phase 1 (median 100.50 s; P25–P75: 75.00–143.50) compared with phase 2 (median 82.00 s; P25–P75: 61.25–118.50) (Wilcoxon test: *p* < 0.001). For cancer cases, the time required for reporting was 18.74% longer during phase 1 (median 253.5 s; P25–P75: 185.50–346.50) than phase 2 (median 206.00 s; P25–P75: 138.25–322.00) (Wilcoxon test: *p* < 0.001). The median time to report cancer cases was longer than the time for reporting negative cases (2.5 times longer in both phases; MN test: *p* < 0.001).

**Fig. 1:**
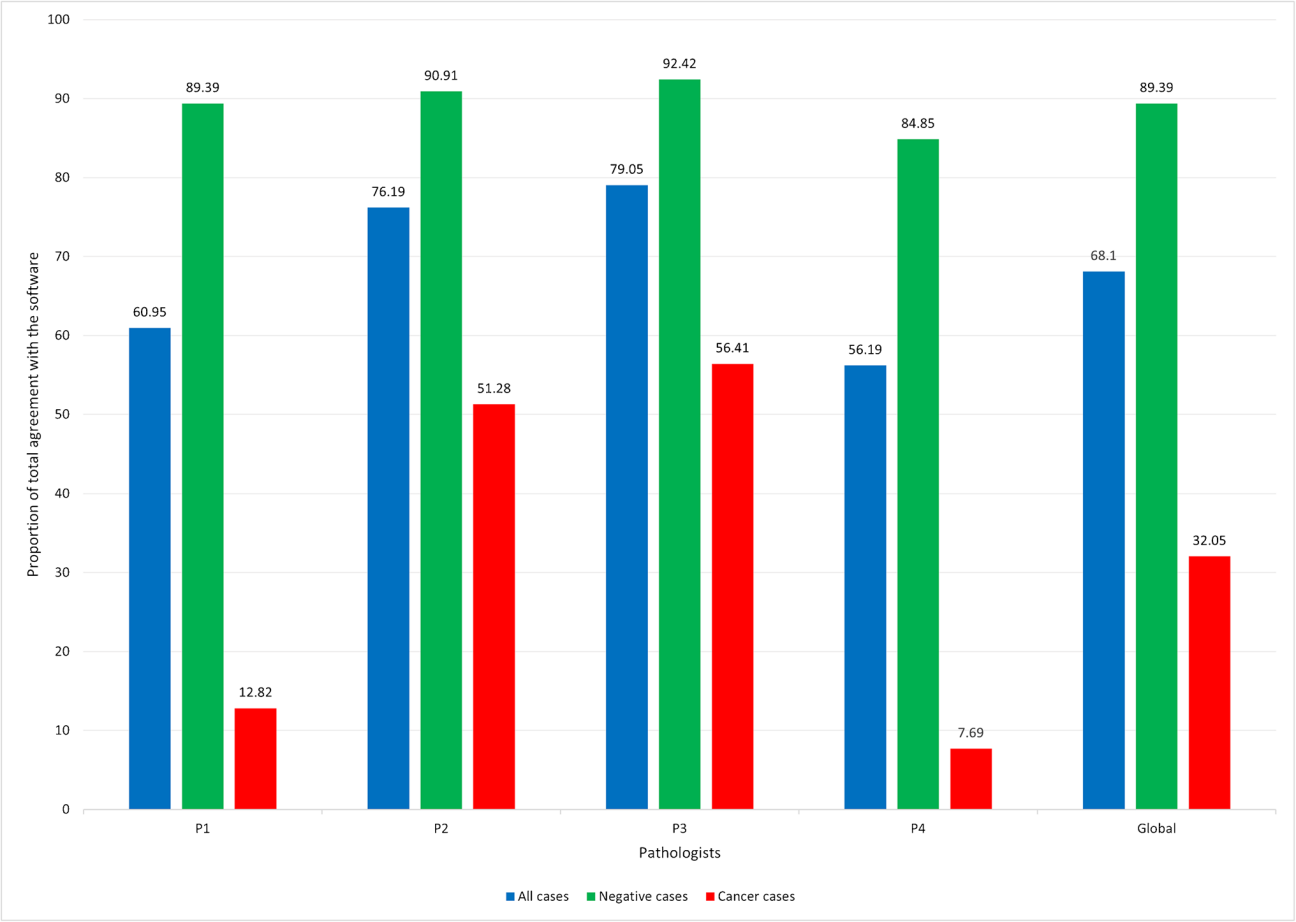
Graphic illustrating the percentage of cases, per pathologist (P1, P2, P3, and P4) and per diagnosis (all cases, only negative cases, and only cancer cases), where the pathologists totally agreed with the software

Lastly, the average total agreement with the software performance was observed in 68.10% of the cases (range: 56.19–79.05%), being significantly higher in negative cases (89.39%; range: 84.85–92.42%) than in cancer cases (32.05%; range: 7.69–56.41%) (chi-squared test: *p* < 0.001, for global evaluation and for each pathologists) (Fig. 1).

**Fig. 2:**
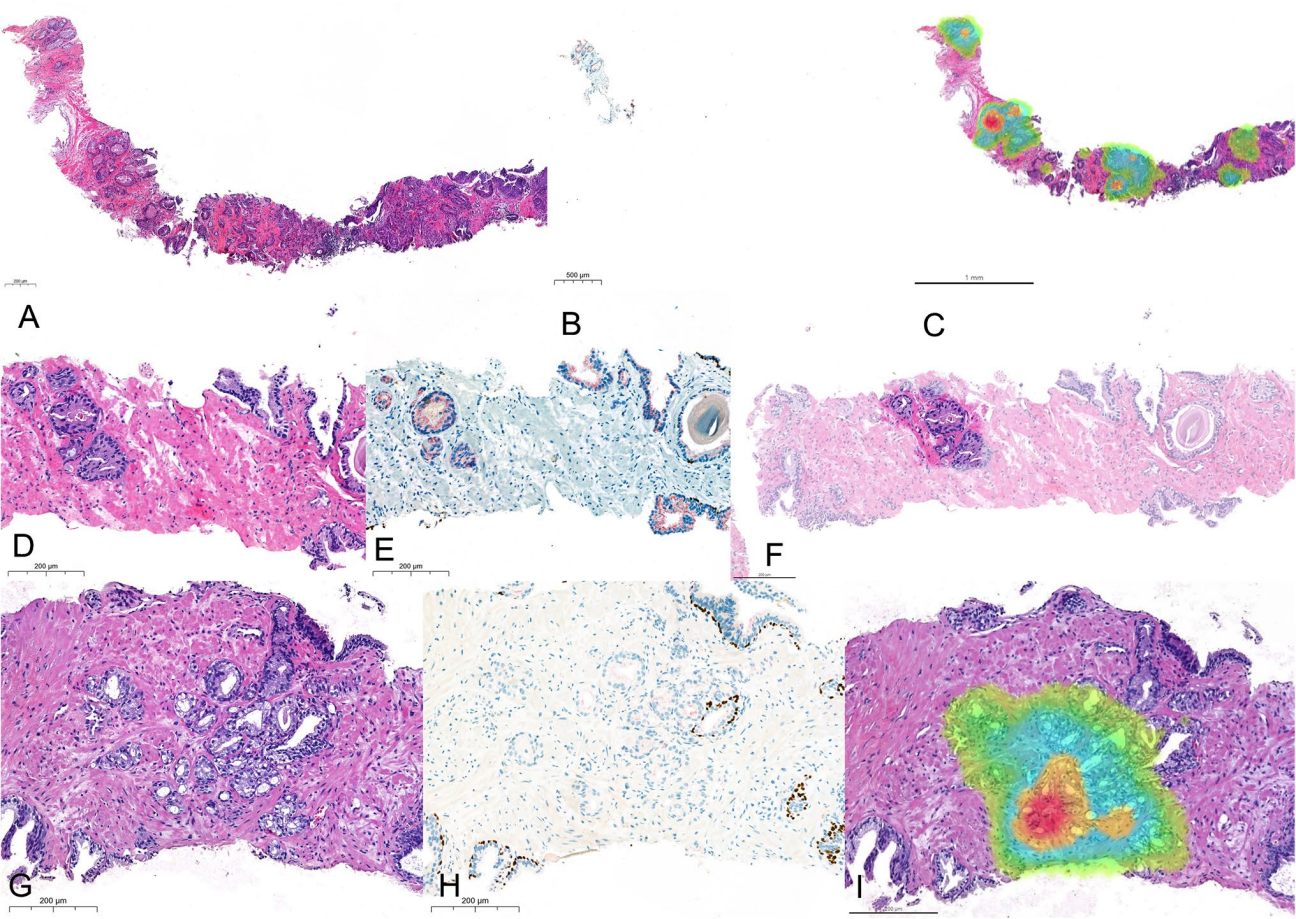
The different sources for observation in 3 cases of the cohort showing for each one of them the HE (**A**, **D**, **G**), IHC with double staining for p63 (brown) and racemase/p504s (red), and the software annotation of cancer (**C** heatmap, **F** shadow, **I** heatmap). Case 33 (**A**–**C**) from an 82-year-old man with acinar adenocarcinoma that lost its representation in IHC slide, contributing to generating discordance among pathologists. Case 57 (**D**–**F**) from a 71-year-old man with a lesion measuring 0.3 mm diagnosed by P1 and P3 as acinar adenocarcinoma GrG1 and by P2 and P4 as ASAP, not changing with the use of the software. Case 77 (**G**–**I**) from a 66-year-old man with a lesion measuring 0.7 mm diagnosed by P3 and P4 as ASAP, changing to acinar adenocarcinoma GrG1 after the use of the software

From the 105 CNB, 92 (87.62%) had perfect intraobserver and interobserver diagnostic concordance. Only 13 cases (12.38%) showed at least one diagnostic discordance in any phase. Most of the diagnostic discordances (10/13; 76.92%) occurred in distinguishing negative cases with ASAP from small foci of well-differentiated (less than 1.5 mm) acinar adenocarcinoma GrG1. The remaining 3 cases corresponded to acinar adenocarcinoma GrG2 with 3.8 mm in which the fragment with cancer was lost in the IHC slide (case 33), acinar adenocarcinoma GrG2 with 0.5 mm (case 61), and an acinar adenocarcinoma post-radiation therapy with 2.3 mm (case 96) (for details, see Table 4 and Fig. 2).

**Table 4:** Cases with discordant diagnosis performed by at least one pathologist in any phase

Case number	Evaluation	Ground truth	P1		P2		P3		P4	
			Phase 1	Phase 2	Phase 1	Phase 2	Phase 1	Phase 2	Phase 1	Phase 2
8	Diagnosis	ASAP	Cancer	Cancer	Cancer	Cancer	ASAP	ASAP	ASAP	ASAP
	GrG	–	GrG1	GrG1	GrG1	GrG1	–	–	–	–
	Lesion size (mm)	0.2	0.2	0.4	1.0	1.0	–	–	–	–
12	Diagnosis	Cancer	Cancer	N	Cancer	Cancer	Cancer	Cancer	Cancer	ASAP
	GrG	GrG1	GrG1	–	GrG1	GrG1	GrG1	GrG1	GrG1	–
	Lesion size (mm)	1.5	1.4	–	1.0	1.5	1.5	1.0	1.0	–
29	Diagnosis	ASAP	N	Cancer	N	ASAP	ASAP	ASAP	ASAP	ASAP
	GrG	–		GrG1		–	–	–	–	–
	Lesion size (mm)	0.8		0.9		–	–	–	–	–
33	Diagnosis	Cancer	Cancer	Cancer	Cancer	Cancer	Cancer	Cancer	ASAP	ASAP
	GrG	GrG2	GrG2	GrG2	GrG2	GrG2	GrG2	GrG2	–	–
	Lesion size (mm)	3.8	4.7	5.8	5.5	4.0	4.9	3.3	–	–
38	Diagnosis	ASAP	Cancer	Cancer	Cancer	Cancer	Cancer	Cancer	ASAP	ASAP
	GrG	–	GrG1	GrG1	GrG1	GrG1	GrG1	GrG1	–	–
	Lesion size (mm)	0.5	0.3	1.0	1.0	1.0	0.8	1.1	–	–
57	Diagnosis	ASAP	Cancer	Cancer	ASAP	ASAP	Cancer	Cancer	ASAP	ASAP
	GrG	–	GrG1	GrG1	–	–	GrG1	GrG1	–	–
	Lesion size (mm)	0.3	0.9	0.4	–	–	1.1	0.4	–	–
61	Diagnosis	Cancer	Cancer	Cancer	ASAP	N	Cancer	Cancer	ASAP	ASAP
	GrG	GrG2	GrG1	GrG3	–		GrG1	GrG2	–	–
	Lesion size (mm)	0.5	0.3	0.5	–		0.7	1.8	–	–
70	Diagnosis	ASAP	Cancer	Cancer	Cancer	Cancer	Cancer	Cancer	ASAP	ASAP
	GrG	–	GrG1	GrG2	GrG3	GrG1	GrG1	GrG1	–	–
	Lesion size (mm)	1.5	1.6	1.5	2.0	3.0	1.6	3.3	–	–
77	Diagnosis	ASAP	Cancer	Cancer	N	ASAP	ASAP	Cancer	ASAP	Cancer
	GrG	–	GrG1	GrG1		–	–	GrG1	–	GrG1
	Lesion size (mm)	0.7	2.2	1.8		–	–	2.5	–	3.0
96	Diagnosis	Cancer*	Cancer	Cancer	ASAP	ASAP	Cancer	Cancer	Cancer	Cancer
	GrG	–	GrG4	GrG5	–	–	GrG5	GrG5	GrG3	GrG4
	Lesion size (mm)	2.3	6.8	8.4	–	–	7.5	7.4	8.0	8.0
103	Diagnosis	Cancer	Cancer	Cancer	Cancer	Cancer	Cancer	Cancer	ASAP	ASAP
	GrG	GrG1	GrG1	GrG2	GrG1	GrG2	GrG1	GrG2	–	–
	Lesion size (mm)	0.6	0.6	0.7	0.6	0.5	0.6	0.7	–	–
104	Diagnosis	ASAP	Cancer	Cancer	ASAP	ASAP	Cancer	Cancer	ASAP	ASAP
	GrG	–	GrG1	GrG1	–	–	GrG1	GrG1	–	–
	Lesion size (mm)	0.5	0.7	0.7	–	–	1.0	0.7	–	–
105	Diagnosis	ASAP	Cancer	Cancer	Cancer	Cancer	Cancer	Cancer	ASAP	ASAP
	GrG	–	GrG1	GrG1	GrG1	GrG1	GrG1	GrG1	–	–
	Lesion size (mm)	0.6	1.0	1.0	1.6	1.0	1.0	1.0	–	–

En dash means not applicable/not reported

*ASAP* atypical small acinar proliferation, *GrG* grade group, *N* negative

^*^Status post radiotherapy

The median size of the biopsy tissue fragments measured by the pathologists was similar in both phase 1 (median 71.0 mm; P25–P75: 50.0–88.7) and phase 2 (median 71.0 mm; P25–P75: 50.0–90.0) (MW test: *p* = 0.776). There was no significant differences in tissue fragment measurements between pathologists in each phase (phase 1 [KW test: *p* = 0.938] and phase 2 [KW test: *p* = 0.798]).

The median size of cancer measured by the pathologists was also similar in phase 1 (median 4.0 mm; P25–P75: 1.9–11.4) in comparison to phase 2 (median 4.0 mm; P25–P75: 2.0–10.1) (MW test: *p* = 0.810). There was no significant differences in cancer size measurements between pathologists in each phase (phase 1 [KW test: *p* = 0.298] and phase 2 [KW test: *p* = 0.217]).

**Fig. 3:**
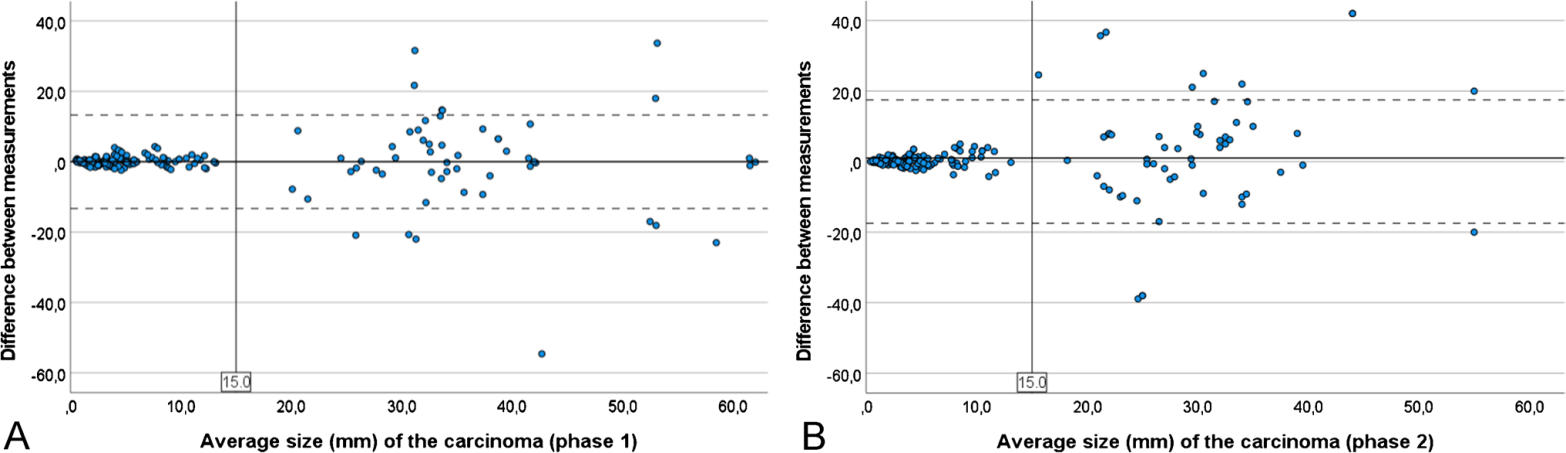
Altman-Bland analysis of the mean difference in cancer size measurements by pathologists in phase 1 (**A**) and phase 2 (**B**), highlighting the very low variability of measurements in low-sized cancers and increased variability in cancer size measurements above 15 mm

The Altman-Bland analysis showed that the mean difference in cancer size measurements between pathologists was 0.07 mm (± 6.76 mm) in phase 1, increasing to 1.02 mm (± 8.93 mm) in phase 2 (Wilcoxon test: *p* = 0.100). However, the variability of the difference in cancer size measurements between pathologists was narrow in cases with cancer smaller than 15 mm (mean difference of 0.12 mm [± 1.11 mm] in phase 1 and 0.25 mm [± 1.47 mm] in phase 2) and wider in cases with cancer greater than 15 mm (mean difference of − 0.08 mm [± 13.97 mm] in phase 1 and 3.16 mm [± 17.07 mm] in phase 2) (Fig. 3A, B).

## Discussion

Previous evidence of the performance of Paige Prostate software has been demonstrated by other independent groups [11, 12]. The study by Perincheri et al. [11] described Paige Prostate software as a good prescreening tool and as a second read tool, contributing to the increase in cancer detection (sensitivity of 97.7%, specificity of 99.3%, positive predictive value of 97.9%, and negative predictive value of 99.2%) and saving time after testing a large series of 1876 prostate CNBs, in a study designed to compare the performance of the software versus the pathologist. The group of da Silva et al. [12] confirms the incremental improvements in diagnostic performance (sensitivity of 0.99, specificity of 0.93, and negative predictive value of 1.00) and describes a possible 65.5% reduction in the diagnostic time after testing a series of 600 CNBs, in a study designed to compare the performance of the software versus the pathologist.

The current study was designed to assess how Paige Prostate, an AI tool trained to detect, grade, and quantify acinar adenocarcinoma, but no other types of cancer, impacts pathologist’s performance. This tool, as per the FDA authorization, is not intended to be used for autonomous diagnosis, but used in synergy with the pathologist. After comparing the stand-alone diagnosis performance with the diagnosis of prostate cancer assisted by Paige Prostate, we report similar high levels of performance, such as those mentioned above. Eventual differences in these values between studies may reflect the influence of the pathologist’s experience and opinion over the software diagnosis that, according to the different degrees of confidence, may interfere with the final diagnosis. The individual motivation of the pathologist to trust the software or not is clearly expressed in Fig. 1, where P4 clearly has a lack of confidence in the software performance and P3 is the pathologists that most frequently agrees with the software, regardless the presence or absence of cancer in the WSI. Reasons for this individual motivation are diverse but include previous habits of the pathologist. The four pathologists participating in this study have worked digitally and routinely for 2 years on the CaseViewer platform from 3DHISTECH and are highly confident in digital pathology for primary diagnosis which was appropriately validated for clinical usage in our laboratory [6]. This is demonstrated by the high mean interobserver diagnostic concordance and diagnostic accuracy in phase 1 (about 95%) [6, 16].

In comparison with the studies by da Silva et al. [12] and Perincheri et al. [11], our series is much smaller, comprising only 105 WSIs that needed IHC studies during primary reporting. This fact may have contributed to concentrate difficult cases in the series, with a higher number of discordant cases (about 12%) and ASAP diagnosis (about 8%) than that described in the literature (1.6–5.8%) [17, 18]. With the help of Paige Prostate, the number of ASAPs significantly decreased (about 30%), indicating that the synergic use of Paige Prostate may contribute to decreasing the level of uncertainty among pathologists. There was no significant impact during the AI-assisted phase in the evaluation of other relevant features such as cribriform pattern, intraductal carcinoma, or perineural invasion, indicating that AI tools directed at supporting pathologists in the detection of these features may further enhance pathologists’ performance in these aspects. The trend to detect less frequently perineural invasion with the use of FullFocus viewer could reflect the use of the new viewer tool instead of the CaseViewer by 3DHISTECH that is used daily in routine diagnosis.

The paradigmatic example of case 33 illustrated in Fig. 2, where the tissue fragment that contained cancer was lost in deeper cuts for IHC staining, shows the value of using the original H&E WSI, overcoming the problems related with loss of tissue. Since tissue loss in deeper cuts is a frequent event in clinical practice, mainly when we are operating with small and linear tissue fragments, the use of AI tools may bring about significant advantages, including efficiencies around tissue logistics as well as being a source of information for diagnosis.

Our series included only acinar adenocarcinomas, the type of cancer that this AI tool was trained to detect. Regarding the grading of these cancers, there was no significant impact in the concordance among pathologists for GrG evaluation, nor in the concordance with the ground truth, with the introduction of the software. Nevertheless, the important intraobserver variability between phases in the evaluation of GrG demonstrates the difficulties in maintaining the reproducibility of GrG evaluation even with the usage of an AI tool. The difficulties in maintaining the reproducibility may mirror the difficulty of the task, the fragility of the ground truth, and most importantly, the influence of the human factor in an evaluation that results from the synergic usage of AI (and not from AI alone), assumed to be certainly more consistent. These obstacles to reproducibility may eventually be overcome by the design of an explainable software program that predicts clinical evolution based on tumor morphology alone and obtained from H&E-stained WSIs, coupled with clinical outcome information that may eventually also challenge the diagnostic criteria of malignancy, at least in very small lesions.

Of note were the significant time savings obtained during the AI-assisted phase, with a reduction of reading times of about 20% with the help of Paige Prostate, in both benign and malignant cases. An additional factor that may contribute to enhance laboratory efficiency and shorter turnaround times is the significant decrease in IHC and second opinion requests during phase 2. In general, IHC requests were reduced by about 20% (about 25% in cancer cases) while second opinion requests were reduced by about 40%. These data points support the previous assumption that, owing to its high sensitivity and negative predictive value, Paige Prostate works well both as a screening element and as a second-read tool by decreasing the need for second opinion requests.

Finally, a comment on the consistence of tissue measurements and cancer size estimations was similar in both phases. This suggests that the use of different viewers during the different phases had no impact on tumor size evaluation. As in other sorts of measures in pathology, it is evident that the larger the measurement, the larger the variability in its evaluation and this is well illustrated in cancer sizes above 15 mm [19]. The variability of measurements was not reduced during phase 2 for cancer size above 15 mm probably because the software does not allow the interference of the pathologist in the estimated measures. This is to say that, after Paige Prostate produces its linear tumor measurement, the pathologist either accepts it entirely or needs to measure it manually. Paige Prostate would benefit from displaying how it has calculated tumor length measurements, and this lack of flexibility to edit automatic measurements was the main justification for the low agreement with the software in cancer cases for some pathologists, as illustrated in Fig. 1. The average total agreement with the software was observed in about 70% of the cases and only in about 30% of the cancer cases. Although these values seem low for an FDA-approved AI tool, especially in cancer cases, the tool could still be used to increase the efficiency of the pathologist’s workflow, without decreasing their accuracy.

Taking in consideration the results of this study and considering the limitations pointed out in this discussion, we may conclude that the synergic usage of Paige Prostate significantly decreases the time for reporting and the consumption of resources (IHC studies and second opinion requests) and preserving tissue/cancer representation in very small biopsies, while maintaining highly accurate diagnostic standards in prostate cancer. In the setting of cancer diagnosis, time is an important variable, not often controlled in pathology laboratories. Turnaround time has an important impact on health-care costs and on-time/life-saving treatments that are here addressed as a great advantage of the use of artificial intelligence.

## Supplementary information

Below is the link to the electronic supplementary material.

Supplementary file1 (DOCX 18 KB)

## Data Availability

The datasets generated during and/or analyzed during the current study are available from the corresponding author on reasonable request.

## References

[1] Siegel RL, Miller KD, Fuchs HE, Jemal A (2022). Cancer statistics, 2022. CA: Cancer J Clin.

[2] Matoso A, Epstein JI (2019). Defining clinically significant prostate cancer on the basis of pathological findings. Histopathology.

[3] Montironi R, Hammond EH, Lin DW, Gore JL, Srigley JR, Samaratunga H, Egevad L, Rubin MA, Nacey J, Klotz L, Sandler H, Zietman AL, Holden S, Humphrey PA, Evans AJ, Delahunt B, McKenney JK, Berney D, Wheeler TM, Chinnaiyan A, True L, Knudsen B, Epstein JI, Amin MB, College of American Pathologists, International Society of Urological Pathology, Association of Directors of Anatomic and Surgical Pathology (2014). Consensus statement with recommendations on active surveillance inclusion criteria and definition of progression in men with localized prostate cancer: the critical role of the pathologist. Virchows Arch.

[4] Yang C, Humphrey PA (2020). False-negative histopathologic diagnosis of prostatic adenocarcinoma. Arch Pathol Lab Med.

[5] Renshaw AA, Cartagena N, Granter SR, Gould EW (2003). Agreement and error rates using blinded review to evaluate surgical pathology of biopsy material. Am J Clin Pathol.

[6] Eloy C, Vale J, Curado M, Polonia A, Campelos S, Caramelo A, Sousa R, Sobrinho-Simoes M (2021) Digital pathology workflow implementation at IPATIMUP. Diagnostics (Basel) 11. 10.3390/diagnostics11112111 10.3390/diagnostics11112111 PMC8620597 34829458

[7] Fraggetta F, L'Imperio V, Ameisen D, Carvalho R, Leh S, Kiehl TR, Serbanescu M, Racoceanu D, Della Mea V, Polonia A, Zerbe N, Eloy C (2021) Best practice recommendations for the implementation of a digital pathology workflow in the Anatomic Pathology Laboratory by the European Society of Digital and Integrative Pathology (ESDIP). Diagnostics (Basel) 11. 10.3390/diagnostics11112167 10.3390/diagnostics11112167 PMC8623219 34829514

[8] Polonia A, Campelos S, Ribeiro A, Aymore I, Pinto D, Biskup-Fruzynska M, Veiga RS, Canas-Marques R, Aresta G, Araujo T, Campilho A, Kwok S, Aguiar P, Eloy C (2021). Artificial intelligence improves the accuracy in histologic classification of breast lesions. Am J Clin Pathol.

[9] Coudray N, Ocampo PS, Sakellaropoulos T, Narula N, Snuderl M, Fenyo D, Moreira AL, Razavian N, Tsirigos A (2018). Classification and mutation prediction from non-small cell lung cancer histopathology images using deep learning. Nat Med.

[10] Kather JN, Krisam J, Charoentong P, Luedde T, Herpel E, Weis CA, Gaiser T, Marx A, Valous NA, Ferber D, Jansen L, Reyes-Aldasoro CC, Zornig I, Jager D, Brenner H, Chang-Claude J, Hoffmeister M, Halama N (2019). Predicting survival from colorectal cancer histology slides using deep learning: a retrospective multicenter study. PLoS Med.

[11] Perincheri S, Levi AW, Celli R, Gershkovich P, Rimm D, Morrow JS, Rothrock B, Raciti P, Klimstra D, Sinard J (2021). An independent assessment of an artificial intelligence system for prostate cancer detection shows strong diagnostic accuracy. Mod Pathol.

[12] da Silva LM, Pereira EM, Salles PG, Godrich R, Ceballos R, Kunz JD, Casson A, Viret J, Chandarlapaty S, Ferreira CG, Ferrari B, Rothrock B, Raciti P, Reuter V, Dogdas B, DeMuth G, Sue J, Kanan C, Grady L, Fuchs TJ, Reis-Filho JS (2021). Independent real-world application of a clinical-grade automated prostate cancer detection system. J Pathol.

[13] Patel AU, Shaker N, Mohanty S, Sharma S, Gangal S, Eloy C, Parwani AV (2022) Cultivating clinical clarity through computer vision: a current perspective on whole slide imaging and artificial intelligence. Diagnostics (Basel) 12. 10.3390/diagnostics12081778 10.3390/diagnostics12081778 PMC9332710 35892487

[14] Campanella G, Hanna MG, Geneslaw L, Miraflor A, Werneck Krauss Silva V, Busam KJ, Brogi E, Reuter VE, Klimstra DS, Fuchs TJ (2019). Clinical-grade computational pathology using weakly supervised deep learning on whole slide images. Nat Med.

[15] Raciti P, Sue J, Ceballos R, Godrich R, Kunz JD, Kapur S, Reuter V, Grady L, Kanan C, Klimstra DS, Fuchs TJ (2020). Novel artificial intelligence system increases the detection of prostate cancer in whole slide images of core needle biopsies. Mod Pathol.

[16] Pantanowitz L, Sinard JH, Henricks WH, Fatheree LA, Carter AB, Contis L, Beckwith BA, Evans AJ, Lal A, Parwani AV, College of American Pathologists Pathology and Laboratory Quality Center,  (2013). Validating whole slide imaging for diagnostic purposes in pathology: guideline from the College of American Pathologists Pathology and Laboratory Quality Center. Arch Pathol Lab Med.

[17] Prathibha S, Goyal KG, Zynger DL (2018). Initial diagnosis of insignificant cancer, high-grade prostatic intraepithelial neoplasia, atypical small acinar proliferation, and negative have the same rate of upgrade to a Gleason score of 7 or higher on repeat prostate biopsy. Hum Pathol.

[18] Nakai Y, Tanaka N, Miyake M, Hori S, Tatsumi Y, Morizawa Y, Fujii T, Konishi N, Fujimoto K (2017). Atypical small acinar proliferation and two or more cores of high-grade intraepithelial neoplasia on a previous prostate biopsy are significant predictors of cancer during a transperineal template-guided saturation biopsy aimed at sampling one core for each 1 mL of prostate volume. Res Rep Urol.

[19] Polonia A, Eloy C, Pinto J, Braga AC, Oliveira G, Schmitt F (2017). Counting invasive breast cancer cells in the HER2 silver in-situ hybridization test: how many cells are enough?. Histopathology.

