# Supplementary material for: Artificial intelligence–assisted cancer diagnosis improves the efficiency of pathologists in prostatic biopsies
Source: Virchows Arch. 2023 Feb 21;482(3):595–604. doi: 10.1007/s00428-023-03518-5 (PMC10033575; doi:10.1007/s00428-023-03518-5)
Supplement: Supplementary file 1 — Supplementary file1 (DOCX 18 KB) [file 428_2023_3518_MOESM1_ESM.docx]

| **Table S1** Interobserver diagnostic concordance (phase 1 and phase 2) | | | | | | | | |
| --- | --- | --- | --- | --- | --- | --- | --- | --- |
|  | Phase 1 | | | | Phase 2 | | | |
|  | P1 | P2 | P3 | P4 | P1 | P2 | P3 | P4 |
| P2 | 95.24%  (0.902) |  |  |  | 93.33%  (0.863) |  |  |  |
| P3 | 98.10%  (0.961) | 95.24%  (0.901) |  |  | 97.14%  (0.942) | 94.29%  (0.882) |  |  |
| P4 | 90.48%  (0.802) | 93.33%  (0.857) | 92.38%  (0.839) |  | 90.48%  (0.802) | 91.43%  (0.816) | 91.43%  (0.821) |  |
| Ground truth | 93.33%  (0.862) | 94.29%  (0.879) | 95.24%  (0.901) | 97.14%  (0.938) | 91.43%  (0.823) | 94.29%  (0.879) | 94.29%  (0.881) | 95.24%  (0.896) |
| Proportion (kappa) | | | | |  |  |  |  |

| **Table S2** Sensitivity, specificity, PPV and NPV in both phases | | | | | | | | |
| --- | --- | --- | --- | --- | --- | --- | --- | --- |
|  | Sensitivity | | Specificity | | PPV | | NPV | |
|  | Phase 1 | Phase 2 | Phase 1 | Phase 2 | Phase 1 | Phase 2 | Phase 1 | Phase 2 |
| P1 | 1.000 | 0.974 | 0.894 | 0.879 | 0.848 | 0.826 | 1.000 | 0.983 |
| P2 | 0.949 | 0.949 | 0.939 | 0.939 | 0.902 | 0.902 | 0.969 | 0.969 |
| P3 | 1.000 | 1.000 | 0.924 | 0.909 | 0.886 | 0.867 | 1.000 | 1.000 |
| P4 | 0.923 | 0.897 | 1.000 | 0.985 | 1.000 | 0.972 | 0.957 | 0.942 |
| Average | 0.968 | 0.955 | 0.939 | 0.928 | 0.909 | 0.892 | 0.982 | 0.974 |
| PPV, positive predictive value; NPV, negative predictive value. | | | | | | | | |

| **Table S3** Interobserver grade group concordance (phase 1 and phase2) | | | | | | |  |  |
| --- | --- | --- | --- | --- | --- | --- | --- | --- |
|  | Phase 1 | | | | Phase 2 | | | |
|  | P1 | P2 | P3 | P4 | P1 | P2 | P3 | P4 |
| P2 | 80.49%  (0.888) |  |  |  | 67.50%  (0.845) |  |  |  |
| P3 | 72.72%  (0.923) | 57.50%  (0.823) |  |  | 72.72%  (0.856) | 80.00%  (0.938) |  |  |
| P4 | 77.78%  (0.942) | 65.71%  (0.874) | 86.11%  (0.942) |  | 69.44%  (0.760) | 64.71%  (0.869) | 77.78%  (0.886) |  |
| Consensus | 76.32%  (0.934) | 64.86%  (0.814) | 86.84%  (0.939) | 94.29%  (0.963) | 67.57%  (0.791) | 72.97%  (0.902) | 86.84%  (0.960) | 88.24%  (0.872) |
| Proportion (kappa quadratic weighted) | | | | |  |  |  |  |
